# Case Report: A thrombosis of ductus arteriosus aneurysm involving the left pulmonary artery in a full-term newborn with isolated right ventricular hypoplasia

**DOI:** 10.3389/fped.2025.1624029

**Published:** 2025-09-25

**Authors:** Stasa Krasic, Nevena Djorovic, Ivan Dizdarevic, Vesna Topic, Nikola Ilic, Adrijan Sarajlija, Dragana Aleksic, Vladislav Vukomanovic

**Affiliations:** ^1^ Cardiology Department, Mother and Child Health Institute of Serbia, Belgrade, Serbia; ^2^ Faculty of Medicine, University of Belgrade, Belgrade, Serbia; ^3^ Cardiac Surgery Department, Mother and Child Health Institute of Serbia, Belgrade, Serbia; ^4^ Radiology Department, Mother and Child Health Institute of Serbia, Belgrade, Serbia; ^5^ Clinical Genetics Outpatient Clinic, Mother and Child Health Institute of Serbia, Belgrade, Serbia; ^6^ Hematology Department, Mother and Child Health Institute of Serbia, Belgrade, Serbia

**Keywords:** ductus arteriosus aneurysm, pulmonary artery thrombus, isolated right ventricular hypoplasia, neonates, case report

## Abstract

**Background:**

Thrombosis of ductus arteriosus aneurysm (DAA) is a well-known complication of DAA that can lead to vascular obstruction or thromboembolic events.

**Case report:**

A full-term male newborn presented with isolated right ventricular hypoplasia (IRVH). Follow-up echocardiography at 19 days of life revealed a pedunculated mass, suggesting a thrombus partially obstructing the left pulmonary artery (LPA). The patient remained clinically stable but was admitted to the neonatal intensive care unit for close monitoring. CT and MRI confirmed DAA thrombosis involving LPA. Due to a lack of resolution with conservative treatment, the patient underwent a thrombectomy and resection of the ductus arteriosus (DA). The postoperative course was uneventful, and the follow-up echocardiography showed normalisation of the right ventricular cavity and no residual thrombus.

**Conclusion:**

This case highlights the importance of early detection and investigation in neonates with echocardiographic findings of intrauterine ductus arteriosus closure, stenosis, or DA closure in the first 12 h of life to prevent life-threatening complications.

## Introduction

Ductus arteriosus aneurysm (DAA) is a rare congenital anomaly characterized by focal saccular or diffuse tubular dilatation of the ductus arteriosus with an estimated incidence up to 8.8% in term neonates (1, 2).

The pathophysiology remains incompletely understood, with multiple contributing factors such as reduced intimal cushions, abnormal elastin deposition, mechanical factors as well as certain genetic conditions (1–3).

While many cases with congenital DAA remain asymptomatic with a benign course and spontaneous regression (2, 3), some present with respiratory distress, stridor, feeble cry, as well as systemic and pulmonary hypertension (4–6). These manifestations typically result from complications such as thrombosis, thromboembolism, rupture or compressive effect on adjacent airways or vasculature (1–6).

Among these complications, DAA thrombosis is particularly concerning, as it can lead to vascular obstruction or thromboembolic events, posing significant diagnostic and therapeutic challenges (2). Because intra-arterial thrombus formation affects approximately 30% of symptomatic cases of DAA, the early detection of high-risk DAA is mandatory for curative intervention.

We present an asymptomatic male newborn diagnosed with isolated right ventricular hypoplasia (IRVH) and thrombosis of DAA involving the left pulmonary artery surgically treated without any reported etiology factor.

## Case report

A male full-term infant was born via spontaneous vaginal delivery to a 20-year-old primigravida who had been using calcium channel blockers since 36 weeks of gestation due to threatened preterm labor. Prenatal Doppler ultrasound at that time revealed high resistance in the umbilical artery as well as right heart enlargement.

The newborn weighed 3,530 g at birth, with an Apgar score of 9 at the 1st and 5th minute, respectively. During 6 h of life, the newborn showed signs of mild cyanosis, which did not improve with the use of diffuse oxygen therapy. He required respiratory support via high-flow nasal cannula with FiO_2_ of 50% for the next 48 h, followed by diffuse oxygen therapy for an additional 5 days. Going forward, he remained hemodynamically stable. Echocardiography was performed, and because of excessive trabeculation and prominent papillary muscle of the right ventricle, he was referred to a tertiary referral heart centre at 5 days of life.

**Figure 1:**
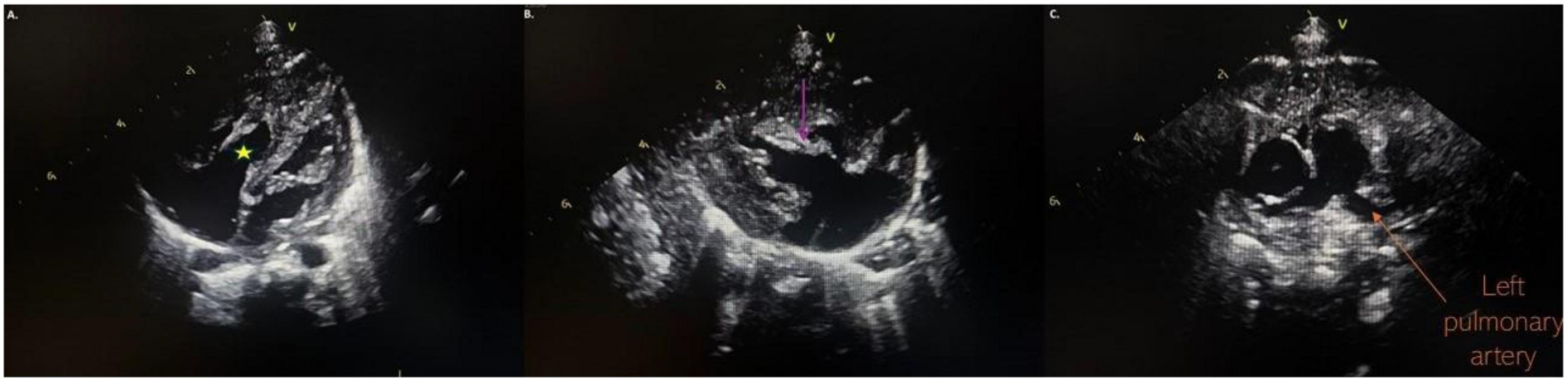
**(A)** Hypertrabecular right ventricle with a small cavity (yellow star); **(B)** massive hyperechogenic tricuspid papillary muscle (purple arrow); **(C)** normal pulmonary artery without pathological masses.

At our hospital, the physical examination was unremarkable. Echocardiography displayed an undeveloped trabecular part of the right ventricle (RV) with a small cavity and prominent papillary muscles (Figure 1A), patent foramen ovale (PFO) with bidirectional, predominantly left to right shunt and mild tricuspid valve regurgitation (peak pressure gradient 45 mmHg), as well as insignificant regurgitation on the mitral valve. Left pulmonary artery (LPA) blood flow was laminar with a velocity of 1.6 m/s (Figure 1). The flow in patent ductus arteriosus (PDA) was not identified. Based on these findings, a diagnosis of IRVH was made, and further monitoring was advised.

**Figure 2:**
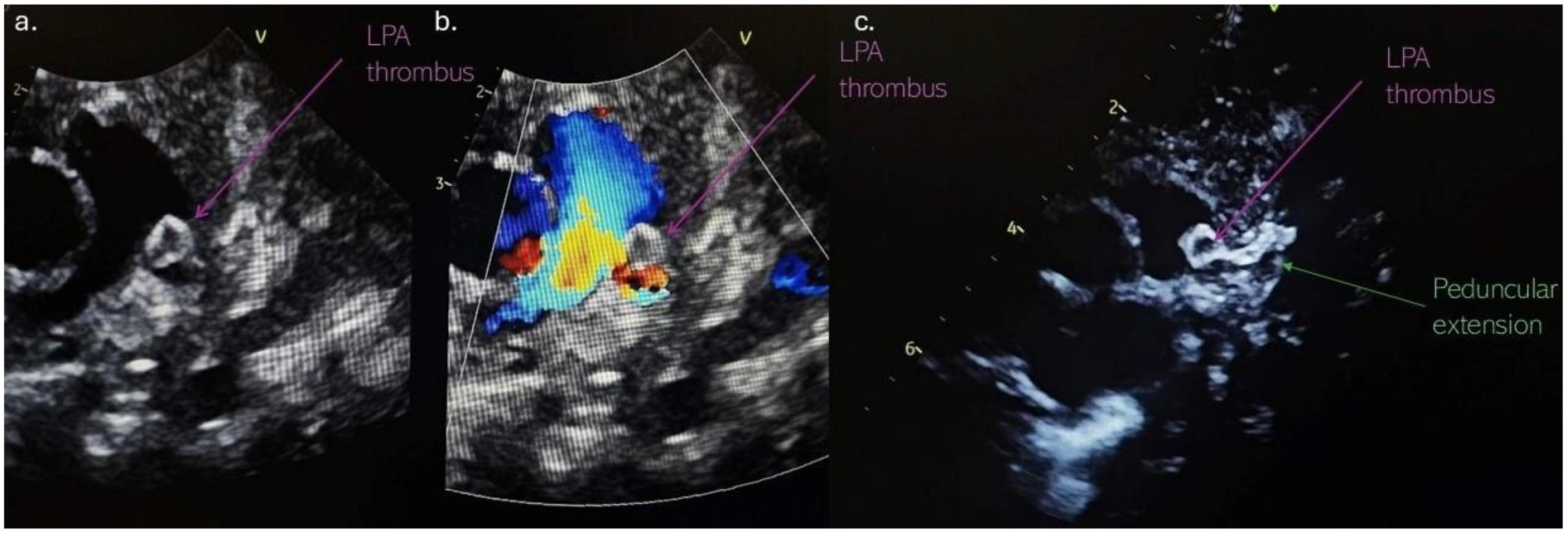
**(a)** Thrombus localised in the left pulmonary artery; **(b)** partially LPA obstruction with an accelerated blood flow in the LPA; **(c)** the thrombus attached to a hyperechoic, tortuous peduncle, suspected to originate from a DA. LPA, left pulmonary artery; DA, ductus arteriosus.

At the follow-up examination 2 weeks later, echocardiography revealed a pedunculated mass 5 mm × 5 mm suggestive of a thrombus, partially obstructing LPA with a pressure gradient of 30 mmHg. The mass was attached to a hyperechoic, tortuous peduncle, suspected to originate from a PDA, with a length of 11 mm (Figure 2). Due to these findings, the infant was admitted to the neonatal intensive care unit department. Upon admission, he remained clinically stable, with SpO_2_ of 100% and no signs of respiratory distress or cyanosis.

His initial laboratory findings were all within the normal range (D-Dimer levels were normal <240 ng/ml). Further analysis of hypercoagulable workup, genetics and screening for thrombophilia all came back negative. The results of genetic testing for connective tissue diseases were also negative. Low molecular weight heparin was initiated, with doses adjusted according to factor Xa levels.

**Figure 3:**
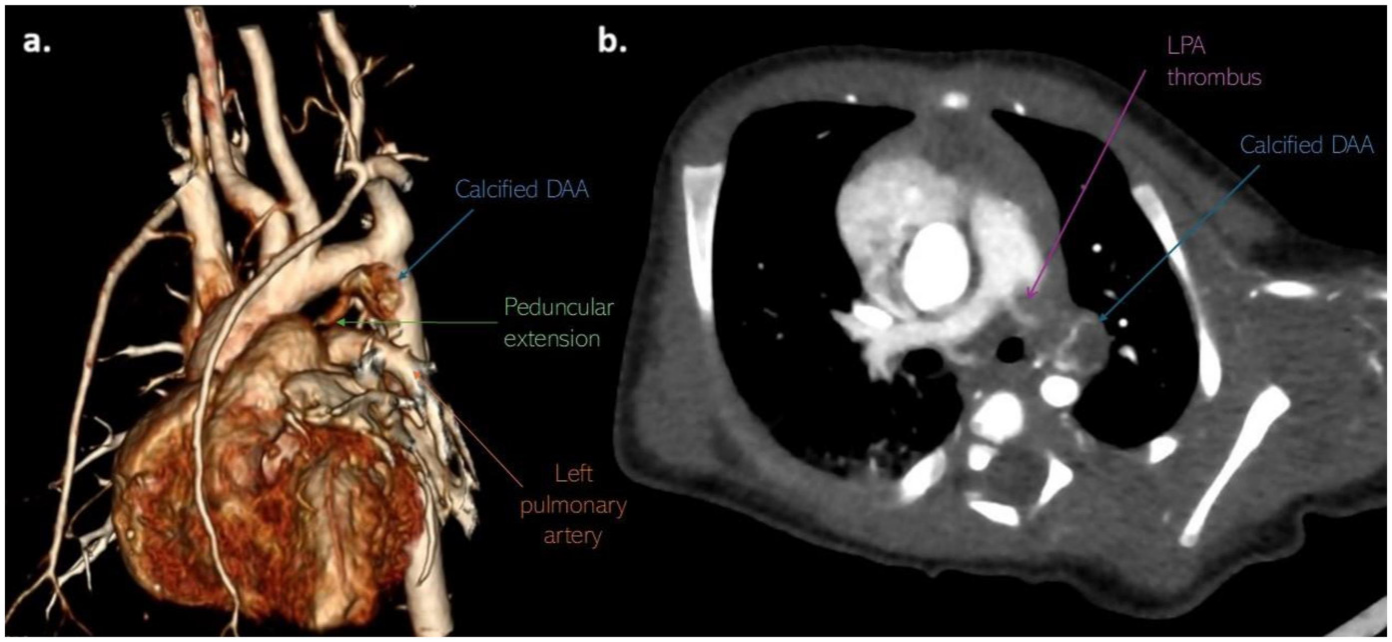
Non-gated CT angiography with **(a)** volume-rendered and **(b)** axial-oblique reconstructions show marginally calcified DAA (blue arrow) with peduncular extension (green arrow) toward the LPA, as well as thrombotic mass (purple arrow) in LPA. CT, computerized tomography; DAA, ductus arteriosus aneurysm; LPA, left pulmonary artery.

Computed tomography (CT) of the chest with angiography revealed an intraluminal filling defect in the proximal LPA measuring 6 mm × 5 mm, resulting in partial obstruction. A marginally calcified structure was also identified in the middle mediastinum, measuring 13 mm × 6 mm × 8 mm, with a ribbon-like calcified tract extending toward the LPA (Figure 3). No evidence of right heart strain was observed.

Additionally, cardiac magnetic resonance imaging (MRI) revealed the lesion along with its previously described location and extent. Based on the characteristics of the MR signal, the most probable differential diagnosis was a DAA with an organised thrombus. Furthermore, this examination demonstrated a small cavity and excessive trabeculation of the right ventricle (RV).

**Figure 4:**
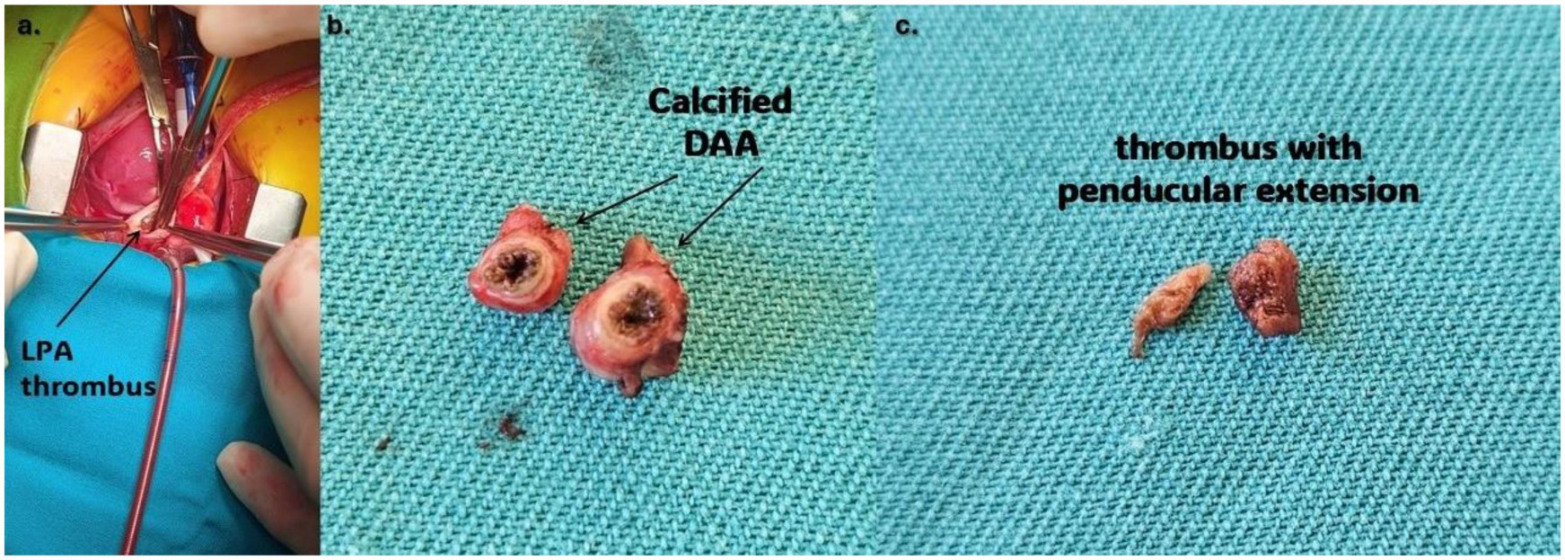
**(a)** Thrombus protruding from aneurysmatically dilated DA (purple arrow); **(b)** resected aneurysm of PDA; **(c)** thrombus that partially occluded left branch of the pulmonary artery. LPA, left pulmonary artery; DA, ductus arteriosus; DAA, ductus arteriosus aneurysm.

Since there was no resolution of the thrombus, the infant underwent thrombectomy and DAA resection. The operation was performed through a median sternotomy (Figure 4a). Dissection of the pulmonary artery confirmed a thrombus with an approximate diameter of 5 mm, partially occluding the left pulmonary artery (Figure 4b). Dissection of the ductus arteriosus revealed a ductal lumen filled with a thrombotic mass and a dilated aortic end measuring 7–8 mm (Figure 4b). The patient was weaned from cardiopulmonary bypass uneventfully. Histopathological examination showed normal ductal tissue with organised thrombus, focusing on hemosiderin and calcification.

The patient had an uneventful postoperative course and was discharged 7 days after surgery without complications. The follow-up echocardiography revealed no valvular abnormalities, normal systolic function, and normalisation of the RV cavity.

## Discussion

The etiopathogenesis of DAA remains unclear, and multiple theories have been proposed to explain it. One is that impairment in forming the ductal intimal cushion, or defective elastin synthesis, may predispose the ductus to aneurysmal transformation, affecting its normal remodelling and closure (7). Another one attributes DAA formation to an inherent fragility of the ductal wall. This may occur from necrosis and mucoid degeneration of the tunica media, leading to structural compromise and aneurysmal dilatation (3, 8). In the case presented by Inagi et al., with a rapidly growing thrombus from the DAA, histological findings showed partial rupture in the DAA, which supports the contention that congenital weakness of the ductal wall contributes to the pathogenesis (9, 10).

In addition to structural and hemodynamic factors, genetic conditions associated with connective tissue abnormalities have been related to the development of DAA (3, 7, 11). Syndromes such as Marfan, Smith-Lemli-Opitz, and Ehlers-Danlos syndrome, all characterised by defects in extracellular matrix components, may contribute to ductal wall weakness and aneurysm formation. Whole-exome genome sequencing ruled out that possibility in our patient. Maternal diabetes mellitus (DM) or those born later in gestation have been reported as a nongenetic risk factor of DAA and umbilical artery thrombosis (12). Use of calcium channel blockers, such in our patient, on the other hand, can inhibit the closure of the ductus arteriosus, particularly in premature infants.

Contrary to pathological explanations, some have proposed that DAA may represent a physiological variant rather than a pathological condition. DAA may represent a standard variant of an elongated ductal bump and be part of the typical process of spontaneous ductal closure in full-term neonates (2).

Moreover, some propose that intrauterine ductal constriction at its pulmonary end results in post-stenotic dilatation. This compensatory enlargement could evolve into an aneurysmal structure (13), identified in approximately 1.5%–2.2% in late pregnancy (14). In our case, a fetal echocardiogram at 36 gestational weeks showed right heart enlargement, and on birth, echocardiography revealed IRVH. Given the fact that a cause of IRVH can be premature closure of the ductus arteriosus *in utero* or the first 12 h of life, we speculate that the pathogenesis of DAA in our case was intrauterine constriction/closure of the pulmonary end of the ductus arteriosus (15). Additionally, some authors claimed that premature PDA closure may create a substrate for thrombus formation (16). Similar to the case of Shirozu et al., our patient had a bidirectional shunt on the PFO due to a restrictive small cavity RV, so we scheduled an early check-up. On the 19th day of life, the newborn was symptom-free, but an echocardiography examination pointed out LPA thrombosis with a tortuous peduncle, suspected to originate from a PDA (12). Firstly, we thought about spontaneous neonatal pulmonary artery thrombosis, which should be suspected in neonates without any cause for cyanosis and respiratory distress (17). Our patient did not have any risk factors for spontaneous neonatal pulmonary artery thrombosis, such as an intravascular catheter, dehydration, sepsis, maternal diabetes, polycythemia, and D-dimers were in the normal range (17, 18). On the other hand, Laviolette et al. presented the case of spontaneous neonatal LPA thrombosis without risk factors, but the prolonged rupture of membrane for 7 days was noted (16). However, CT and MRI examinations of our patient pointed out DAA thrombosis involving LPA.

The thrombosis of DAA involving the pulmonary artery is a well-documented complication, particularly in neonates (3–5, 9, 19). Shirozu et al. presented their case of DAA and pulmonary artery thrombosis in a protein S-deficient newborn with a comprehensive literature review and found that only their patient was asymptomatic (12). The diagnosis was made on the median 1st day of life, and the patients were cyanotic and had signs of respiratory distress (12). In some cases, associated conditions and genetic predisposition were described, such as protein S deficiency, umbilical line, prolonged prothrombin time and activated partial thromboplastin time, perinatal asphyxia, maternal diabetes mellitus and heterozygote of methylenetetrahydrofolate reductase (MTHFR) (12, 20). At the same time, none of the predisposing factors were found in our patient.

The management of DAA with thrombosis varies from pharmacological treatment to thrombectomy, depending on clinical manifestations and complications. In our case, definitive surgical management was necessary since there was obstruction of blood flow, no resolution of thrombus with pharmacological treatment, as well as a risk of thromboembolism. Inagi et al. underwent their patient's surgery due to a rapidly growing thrombus in the LPA despite systemic heparinisation (9). In most cases in the literature, surgery was the definitive treatment (5, 12, 14, 25). Xie et al. suggested that when the descending aorta is involved, complete excision of the DAA and the affected arterial wall is crucial to prevent thrombus recurrence (14). On the other hand, Takajo and Kobayashi presented a newborn with DAA and a 1.2 cm × 0.5 cm × 0.5 cm intraluminal PA thrombus that was conservatively treated. At 4 months follow-up, the echogenic mass spontaneously regressed with no evidence of left pulmonary artery stenosis (20). By searching the PubMed database, typing in the keywords: aneurysm of the ductus arteriosus, thrombosis, newborn, several cases were found (Table 1). Additionally, conservative treatment is the first-line treatment in spontaneous neonatal pulmonary artery thrombosis (16–18).

**Table 1:** Literature review and presentation of patients with ductus arteriosus thrombosis.

Case number	Reference	DOL	Clinical signs	Treatment	Associated conditions/Genetic predisposition
1	Fripp et al. (10)	1	Vomit, collapse, hepatomegaly	Surgery	Prolonged PT and APTT
2	Dyamenahalli et al. (3)	1	Heart murmur, cyanosis	Surgery	
3	Nyp et al. (21)	0	Heart murmur, cyanosis	Surgery	Maternal GDM heterozygote of MTHFR C677T
4	Masood et al. (22)	0	Acute respiratory distress	Conservative (Aspirin)	
5	Ciliberti et al. (23)	1	Acute respiratory distress, PH	Conservative (Enoxaparin)	Umbilical line heterozygote of MTHFR
6	McArdle et al. (5)	5	Heart murmur, cyanosis	Surgery	
7	Aly et al. (24)	1	Differential cyanosis	Conservative (enoxaprin) + Surgery	
8	Takajo and Kobayashi (20)	1	Differential cyanosis	Conservative	
9	Inagi et al. (9)	1	Acute respiratory disorders, neonatal asphyxia	Conservative (systemic heparinization) + surgery	
10	Shirozu et al. (12)	7	Symptoms-free	Surgery	Protein S deficiency
11	Xie et al. (14)	9	Symptoms-free, systolic murmur	Surgery	Prolonged rupture of membrane

DOL, day of life; GDM, gestational diabtes mellitus; PT, prothrombin time; aPTT, activated partial thromboplastin time; PH, pulmonary hypertension; MTHFR, methyltetrahydrofolate reductase.

In our patient, DAA and LPA thrombosis with concomitant IRVH were found, and both pathological conditions could be a consequence of intrauterine ductal stenosis or occlusion. Consequently, cardiologists should suspect and investigate this echocardiographic finding in neonates with intrauterine ductus arteriosus closure, stenosis, or PDA closure in the first 12 h of life.

## Data Availability

The original contributions presented in the study are included in the article/Supplementary Material, further inquiries can be directed to the corresponding author.
